# *Arthrobotrys blastospora* sp. nov. (Orbiliomycetes): A Living Fossil Displaying Morphological Traits of Mesozoic Carnivorous Fungi

**DOI:** 10.3390/jof9040451

**Published:** 2023-04-06

**Authors:** Fa Zhang, Saranyaphat Boonmee, Yao-Quan Yang, Fa-Ping Zhou, Wen Xiao, Xiao-Yan Yang

**Affiliations:** 1 Institute of Eastern-Himalaya Biodiversity Research, Dali University, Dali 671003, China; zhangf@eastern-himalaya.cn (F.Z.);; 2 Center of Excellence in Fungal Research, Mae Fah Luang University, Chiang Rai 57100, Thailand; 3 School of Science, Mae Fah Luang University, Chiang Rai 57100, Thailand; 4 Key Laboratory of Yunnan State Education Department on Er’hai Lake Basin Protection and the Sustainable Development Research, Dali University, Dali 671003, China; 5 The Provincial Innovation Team of Biodiversity Conservation and Utility of the Three Parallel Rivers from Dali University, Dali University, Dali 671003, China; 6 Yunling Black-and-White Snub-Nosed Monkey Observation and Research Station of Yunnan Province, Dali 671003, China

**Keywords:** blastospores, fossil fungi, nematode-trapping fungi, Orbiliaceae, relic species, trapping structures

## Abstract

The evolution of carnivorous fungi in deep time is still poorly understood as their fossil record is scarce. The approximately 100-million-year-old Cretaceous *Palaeoanellus dimorphus* is the earliest fossil of carnivorous fungi ever discovered. However, its accuracy and ancestral position has been widely questioned because no similar species have been found in modern ecosystems. During a survey of carnivorous fungi in Yunnan, China, two fungal isolates strongly morphologically resembling *P. dimorphus* were discovered and identified as a new species of *Arthrobotrys* (Orbiliaceae, Orbiliomycetes), a modern genus of carnivorous fungi. Phylogenetically, *Arthrobotrys blastospora* sp. nov. forms a sister lineage to *A. oligospora*. *A. blastospora* catches nematodes with adhesive networks and produces yeast-like blastospores. This character combination is absent in all other previously known modern carnivorous fungi but is strikingly similar to the Cretaceous *P. dimorphus*. In this paper, we describe *A. blastospora* in detail and discuss its relationship to *P. dimorphus*.

## 1. Introduction

The origin and evolution of life are the core of biological research [1]. As primary decomposers in nature, fungi play a vital role in the circulation of matter and energy in ecosystems [2,3,4]. Studying fungal evolution is integral to understanding the origin and evolution of life. In nature, most fungi are saprophytic, symbiotic, and parasitic, while a few fungi feed on micro-animals such as nematodes, rhizopods, rotifers, and mites (carnivorous fungi) [5,6,7,8]. This survival strategy of feeding on other microorganisms is generally considered an adaptive evolution of fungi to allow them to adapt to nitrogen deficiency [8,9]. The study of the origin and evolution of carnivorous fungi is crucial for understanding the history of fungal evolution. More than 80% of carnivorous fungi in the whole fungal kingdom belong to Orbiliaceae (Orbiliomycetes, Ascomycota) [9,10]. These fungi capture nematodes with various trapping structures. Modern molecular phylogenetic and morphological studies have divided all Orbiliomycetes carnivorous species into three genera according to type of trapping structure. *Drechslerella* captures nematodes using constricting rings, *Arthrobotrys* produces adhesive networks, and *Dactylellina* captures nematodes with adhesive branches, adhesive knobs, and non-constricting rings [11,12,13]. Although such studies have revealed the phylogenetic relationship among these fungi, the evolutionary hypothesis of Orbiliomycetes carnivorous fungi is still controversial [9,10,11,13,14,15,16,17].

Fossils hold the key that nature provided us to breakthrough in the study of the origin and evolution of life [18,19,20,21]. However, compared with animals and plants, most fungi are tiny and do not have solid tissue structures to form fossils and be discovered by humans. Therefore, understanding the origin and evolution of fungi is very difficult. Excitingly, a few fossils that might be related to carnivorous fungi have been discovered. Jansson and Poinar found several conidia that resembled modern carnivorous fungi and several nematodes with appendages (the morphology of which is similar to adhesive spores or adhesive knobs produced by carnivorous fungi) attached to their bodies and fungal mycelium in their bodies in approximately 26-million-year-old amber [22]. Schmidt et al. [23,24] discovered the oldest relatively complete and clear fossil of a possible carnivorous fungus (*Palaeoanellus dimorphus*) in approximately 100-million-year-old amber, which caused a stir in the research on carnivorous fungi. Unfortunately, no species similar to this fossil has been found in the modern ecosystem so far. It has therefore been unclear whether this fossil is an ancestor of modern carnivorous fungi [12,23,25] because the character combination found in this fossil was distinct from any extant taxa.

During our large-scale survey of carnivorous fungi in the three parallel rivers region in China, two extraordinary carnivorous fungal isolates were discovered from 8698 carnivorous fungal strains isolated from 3617 soil samples and identified as a new species of the Orbiliomycetes carnivorous fungi. Fascinatingly, the morphological characteristics of this species are different from other modern carnivorous fungi and are strikingly similar to the carnivorous fossil fungus (*Palaeoanellus dimorphus*) discovered by Schmidt [23,24], according to which we speculate that this new species is a relict descendant of *P. dimorphus*. The discovery of this new species suggests that *P. dimorphus* is a possible ancestor of Orbiliomycetes carnivorous fungi and provides more accurate information for the evolutionary study of this group of fungi.

## 2. Materials and Methods

### 2.1. Sample Collection

The two carnivorous fungal strains were isolated from two freshwater sediment samples in the Nujiang River Basin, the core area of the three parallel rivers. The sample numbers were EOS-1 (N 27°43′14.60″, E 98°41′30.20″) and EMS-2 (N 27°24′33.20″, E 98°49′34.70″). The freshwater sediment samples were removed from the water with a Peterson bottom sampler (HL-CN, Wuhan Hengling Technology Company, Limited, Wuhan, China). The samples were placed into zip lock bags and stored at 4 °C until processing.

### 2.2. Fungal Isolation

Three to five g of freshwater sediment sample was sprinkled on the surface of cornmeal agar plates (CMA) with sterile toothpicks. Roughly 5000 nematodes (*Panagrellus redivivus* Goodey, free-living nematodes) were added as bait to induce the germination of carnivorous fungi [12,26]. The plates were incubated at 26 °C for three weeks and then observed under a stereomicroscope. A sterile needle was used to transfer a single spore of carnivorous fungi to fresh CMA plates. This step was repeated until a pure culture was attained [10,12].

### 2.3. Morphological Observation

The pure culture was transferred to fresh potato dextrose agar plates (PDA) using a sterile needle and incubated at 26 °C to observe the color and texture of the colony. The pure culture was transferred to the fresh observation well CMA plates (a 2 × 2 cm observation well was created by removing agar from each plate) using a sterile needle. It was incubated at 26 °C until the mycelium overspread the well. Then, approximately 1000 living nematodes (*P. redivivus*) were added to the well to induce the formation of the trapping structure. The trapping structure in the observation well and the conidiophores extending from the wall of the observation well were photographed with an Olympus BX53 microscope (Olympus Corporation, Tokyo, Japan) and Keyence VHX-6000 super deep scene 3D microscope (Keyence Corporation, Osaka, Japan), respectively. A sterile cover glass was obliquely inserted into the fresh CMA plates. Then, strains were inoculated on the plates at 26 °C. The cover glass was removed after the mycelia covered it and was then placed on the glass slide with 0.3% Melan stain to make a temporary slide [12]. The morphological characteristics of conidia and conidiophores were measured and photographed by an Olympus BX53 microscope (Olympus Corporation, Tokyo, Japan).

### 2.4. DNA Extraction, PCR Amplification, and Sequencing

The strain was inoculated in PDA plates at 26 °C for ten days. The mycelium was collected using a sterile scalpel. A rapid fungal genomic DNA isolation kit (Sangon Biotech, Limited, Shanghai, China) was used to extract the total genomic DNA. The primer pairs ITS4-ITS5 [27], 526F-1567R [28], and 6F-7R [29] were used to amplify the ITS, TEF, and RPB2 regions. The PCR amplification was performed in a 50 μL reaction system (2 uL DNA template, 3 μL 25 mM MgCl2, 5 μL 10 × PCR buffer, 1 μL 10 μM dNTPs, 2 μL each primer, 1 unit Taq Polymerase, and 34 uL ddH2O) under the following PCR conditions: 4 min of pre-denaturation at 94 °C; followed by 35 cycles of denaturation at 94 °C for 45 s; 1 min of annealing at 52 °C (ITS), 55 ˚C (TEF), or 54 °C (RPB2); and 1.5–2 min of extension at 72 °C, with a final extension of 10 min at 72 °C. A DiaSpin PCR Product Purification Kit (Sangon Biotech, Limited, Shanghai, China) was used to purify the PCR products. The purified PCR products of the ITS and RPB2 regions were sequenced in the forward and reverse directions using PCR primers, and the primer pair 247F-609R was used to sequence the TEF gene (BioSune Biotech, Limited, Shanghai, China). SeqMan v. 7.0 [30] was used to check, edit, and assemble the sequences. The sequences generated in this study were deposited in the GenBank database (NCBI, https://www.ncbi.nlm.nih.gov/ (accessed on 2 December 2022)).

### 2.5. Phylogenetic Analysis

The sequences generated in this study were deposited in the NCBI Genbank database (Table 1) and compared against the database using BLASTn (https://blast.ncbi.nlm.nih.gov/ (accessed on 1 December 2022)) to determine the attribution of the new isolates. The ITS, TEF, and RPB2 sequences of all reliable taxa of the corresponding genus and partial taxa of the related genus were downloaded (Table 1) according to the BLASTn search results and relevant publications [12,13,31,32]. Three genes were aligned using the online program MAFFT v.7 (http://mafft.cbrc.jp/alignment/server/ (accessed on 3 December 2022)) [33], manually adjusted using BioEdit v7.2.3 [34], and then linked using MEGA6.0 [35]. *Vermispora fusarina* YXJ02-13-5 was selected as an outgroup. Phylogenetic trees were inferred via maximum likelihood (ML) and Bayesian inference (BI) analyses.

The GTR + I + G, SYM + I + G, and GTR + I + G models were selected as the best-fit optimal substitution models of ITS, TEF, and RPB2, respectively, via jModelTest v2.1.10 [47].

IQ-Tree v1.6.5 [48] was used to implement the maximum likelihood (ML) analysis. The dataset was partitioned, and each gene was analyzed with the corresponding optimal substitution model. The statistical bootstrap support values (BS) were computed using rapid bootstrapping with 1000 replicates [49].

A Bayesian inference (BI) analysis was conducted with MrBayes v. 3.2.6 [50]. Fasta Convert [51] was used to convert the multiple sequence alignment file into a MrBayes-compatible NEXUS file. The dataset was partitioned, and the optimal substitution models of each gene were equivalently replaced to conform to the setting of MrBayes. Six simultaneous Markov chains were run for 10,000,000 generations, and trees were sampled every 100 generations. The first 25% of the trees were discarded, and the remaining trees were used to calculate the posterior probabilities (PP) in the majority rule consensus tree. The above parameters were edited in the MrBayes block in the NEX file.

The trees were visualized with FigTree v1.3.1 [52]. The backbone tree was edited using Microsoft PowerPoint (2013) and Adobe Photoshop CS6 software (Adobe Systems, San Jose, CA, USA).

## 3. Results

### 3.1. Phylogenetic Analysis

**Figure 1:**
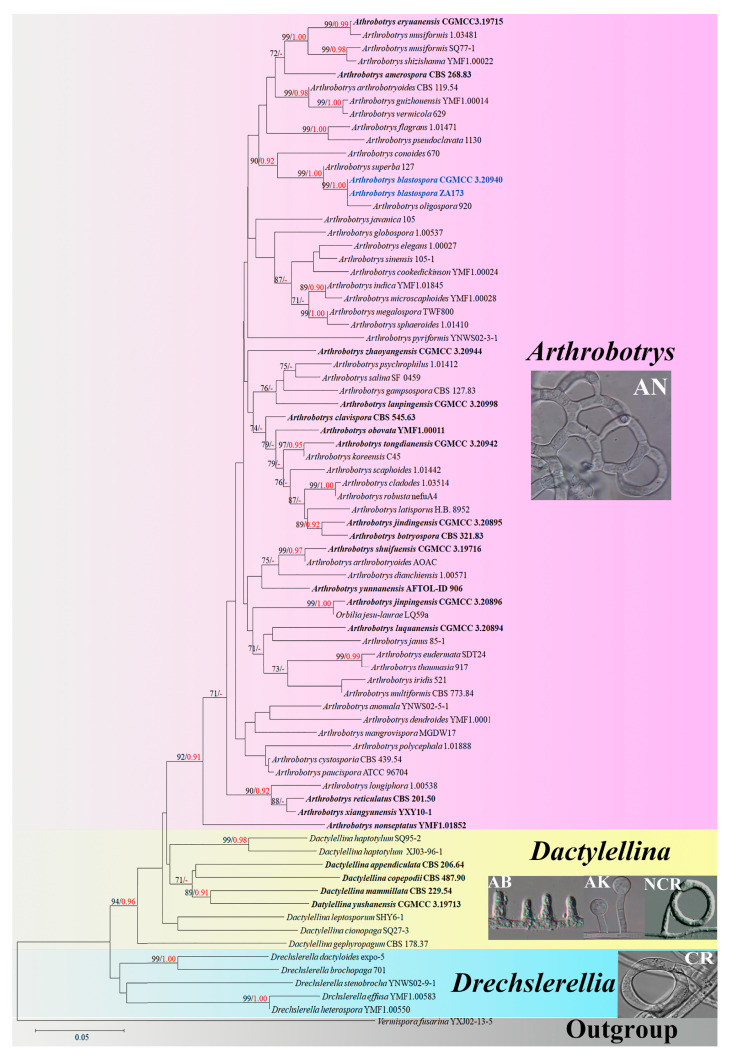
Maximum likelihood tree based on combined ITS, TEF, and RPB2 sequence data from 72 Orbiliaceae (Orbiliomycetes) carnivorous species. Bootstrap support values for maximum likelihoods (black) equal to or greater than 70% and Bayesian posterior probability values (red) equal to or greater than 0.90 are indicated above the nodes. The new isolates are in blue and the type strains are in bold. The genus name and trapping structure corresponding to each clade are indicated on the right. AN, adhesive networks; AB, adhesive branches; AK, adhesive knobs; NCR, non-constricting rings; CR, constricting rings.

Both new fungal isolates were placed in the *Arthrobotrys* (Orbiliaceae, Orbiliomycetes) genus according to their type of trapping structure [11,12,13] and the BLASTn search results of ITS, TEF, and RPB2 genes. Therefore, all *Arthrobotrys* species with valid sequence data (62 isolates representing 59 species) [32] and other related taxa in Orbiliomycetes (9 isolates representing 8 *Dactylellina* species and 5 isolates representing 5 *Drechslerella* species) were included in this phylogenetic analysis (Table 1). The final dataset contained 77 ITS, 51 TEF, and 54 RPB2 sequences. The combined DNA dataset comprised 1909 characters (570 for ITS, 531 for TEF, and 708 for RPB2), among which 858 bp are constant, 982 bp are variable, and 770 bp are parsimony informative. After maximum likelihood (ML) analysis, the best-scoring likelihood tree was obtained with a final ML optimization likelihood value of -6867.586931. The Bayesian analysis (BI) evaluated the Bayesian posterior probabilities with a final average standard deviation of the split frequency of 0.009098. The trees generated by maximum likelihood (ML) and Bayesian analysis (BI) showed similar topologies, so the best-scoring ML tree was selected for presentation (Figure 1).

The phylogenetic analysis showed that the tested 72 Orbiliaceae (Orbiliomycetes) carnivorous species were clustered into three clades according to their types of trapping structure. All species catch nematodes with adhesive networks clustered together stably. Both new fungal isolates were placed in *Arthrobotrys* and formed a basal lineage with *A. oligospora* and *A. superba* with 99% MLBS and 1.00 BYPP (Figure 1).

### 3.2. Taxonomy

**Figure 2:**
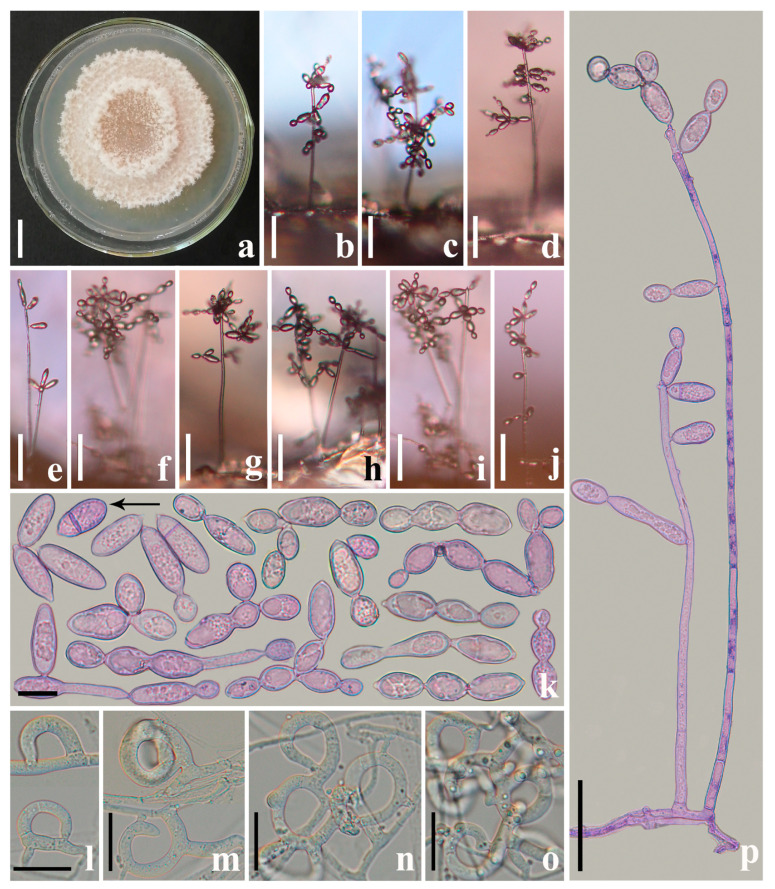
*Arthrobotrys blastospora* (CGMCC 3.20940). (**a**) Colony; (**b**–**j**) Conidiophores and blastospores; (**k**) Blastospores; (**l**–**o**) Trapping structure: adhesive networks; (**p**) Conidiophores. Scale bars: (**a**) = 1 cm; (**b**–**j**) = 10 μm; (**k**–**o**) = 20 μm; (**p**) = 100 μm.

**Figure 3:**
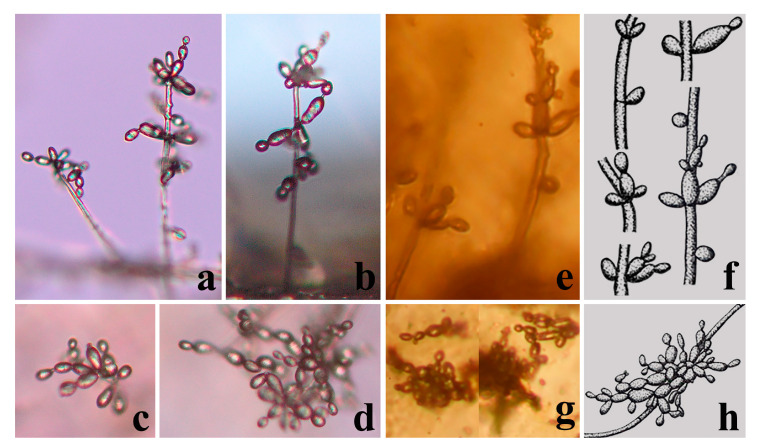
Blastospores and conidiophores of fossil and extant carnivorous fungi. (**a**,**b**) Conidiophores of *Arthrobotrys blastospora*; (**c**,**d**) Blastospores of *Arthrobotrys blastospora*; (**e**,**f**) Conidiophores of the fossil *Palaeoanellus dimorphus*; (**g**,**h**) Blastospores of the fossil *Palaeoanellus dimorphus,* reprinted with permission from Ref. [24] 2023, John Wiley and Sons.

**Figure 4:**
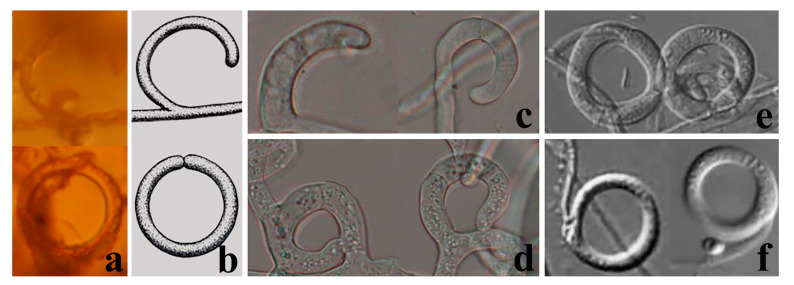
Trapping structure of fossil and extant carnivorous fungi. (**a**,**b**) Trapping structure of *Palaeoanellus dimorphus*: unicellular adhesive hyphal rings, reprinted with permission from Ref. [24]. 2023, John Wiley and Sons; (**c**,**d**) The early stages of adhesive networks produced by *Arthrobotrys blastospora*: single adhesive hyphal rings; (**e**,**f**) Trapping structure of some *Dactylellina* species: non-constricting rings [12].

**Figure 5:**
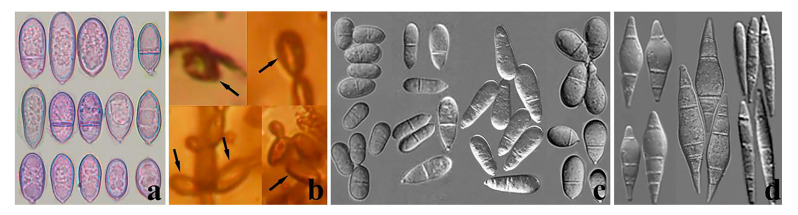
Conidia of several carnivorous fungi. (**a**) The single conidia formed by the separation of the blastospore of *Arthrobotrys blastospora*; (**b**) The single conidia produced by *Palaeoanellus dimorphus*, reprinted with permission from Ref. [24]. 2023, John Wiley and Sons; (**c**) The conidia produced by some *Arthrobotrys* species [31]; (**d**) The conidia produced by most of the *Dactylellina* species [31].

*Arthrobotrys blastospora* F. Zhang and X.Y. Yang sp. nov. (Figure 2, Figure 3a, Figure 4b and Figure 5a).

Index Fungorum number: IF900162; Facesoffungi number: FoF 14034

Etymology: The species name “blastospora” refers to the most prominent feature of this species, i.e., the production of blastospores.

Materials examined: CHINA, Yunnan Province, Nujiang City, Nujiang River, N 27°43′14.60″, E 98°41′30.20″, from freshwater sediment, 18 May 2014, F. Zhang. Holotype CGMC 3.20940, preserved in the China General Microbiological Culture Collection Center. Ex-type culture DLUCC 27-1, preserved in the Dali University Culture Collection.

*Colonies* white, cottony, and rapidly growing on the PDA medium, reaching 50 mm diam after 7 days at 26 °C. *Mycelium* 2.5–6 µm wide, hyaline, septate, branched, and smooth. *Conidiophores* 110–625 µm ($\overset{-}{x}$ = 341.5 µm, *n* = 100) long, 4–6.5 µm ($\overset{-}{x}$ = 5 µm, *n* = 100) wide at the base, gradually tapering upwards to a width of 3–6 µm ($\overset{-}{x}$ = 4 µm, *n* = 100) at the apex, hyaline, erect, septate, unbranched, produced by hyphae or directly by spore germination. This species produces hyaline, yeast-like *blastospores*, which usually cluster on the tuberculous bulges on the upper half of the conidiophores. A conidium is produced on the conidiophore first; then, a second conidium is formed from the right apex, or occasionally the side apex, of the first conidium, thus continuously producing a chain of blastospores. There is a septum between the two conidia, which tend to separate from each other after maturation. *The exfoliated blastospores* 13.5 − 62.5 × 7.5 − 16 ($\overset{-}{x}$ = 25.6 × 10.5 μm, *n* = 300) µm, globose, and elliptic to long elliptic, with zero or one septum. *Chlamydospores* not observed. Capturing nematodes with the adhesive networks in the early stages of its formation usually consists of a single adhesive hypha ring (Figure 2) [12].

Additional specimens examined: CHINA, Yunnan Province, Nujiang City, Nujiang River, N 27°24′33.20″, E 98°49′34.70″, from freshwater sediment, 20 May 2014, F. Zhang. Living culture ZA173.

## 4. Discussion

### 4.1. New Species of Arthrobotrys

*Arthrobotrys blastospora* catches nematodes with adhesive networks, which is consistent with the main characteristics of *Arthrobotrys*, Orbiliaceae (the largest genus of modern carnivorous fungi) [11,12,13,25]. Our phylogenetic analysis based on ITS, TEF, and RPB2 substantiated that *A. blastospora* is a member of *Arthrobotrys*. Phylogenetically, *A. blastospora* forms the sister lineage to *A.oligospora* (Figure 1). However, the conidia of all modern carnivorous fungi in Orbiliomycetes are individually born in clusters or singly on the conidiophores, which is significantly different from the catenulate blastospores produced by *A. blastospora* [12,31]. Therefore, we identified *A. blastospora* as a new species of *Arthrobotrys*.

### 4.2. Palaeoanellus Dimorphus Is an Ancient Ancestor of Modern Orbiliomycetes Carnivorous Fungi

Similar to carnivorous plants, as a highly specialized group in the fungal kingdom, carnivorous fungi are a model for the study of adaptive fungal evolution; their evolution is also a critical node in the study of fungal evolution. Such studies rely heavily on the discovery of fossil fungi. Schmidt et al. [23,24] found the earliest and best-preserved fossil of carnivorous fungi (*P. dimorphus*) in approximately 100-million-year-old amber from Southwestern France. *P. dimorphus* produced blastospores which were generated in whorls on small projections of conidiophores and also produced unicellular adhesive hyphal rings to trap nematodes (Figure 3 and Figure 4) [23,24]. However, no structure directly connecting the blastospores to unicellular adhesive hyphal rings was illustrated by Schmidt et al. [23,24]. Therefore, whether the blastospores and unicellular adhesive hyphal rings in this fossil actually represent a single fossil species has been a controversial topic due to their unusual combination [53,54]. In addition, the fossil was not considered an ancestor of modern carnivorous fungi but as belonging to an extinct lineage because no blastospore-producing carnivorous fungi were found in modern ecosystems [12,23,24,25]. *A. blastospora* reported in this study produced yeast-like blastospores on the small projections of conidiophores and captured nematodes with adhesive networks (a single adhesive hypha ring structure at the initial stage of its formation) (Figure 2) [31]. The discovery of this species confirms the existence of an extant species in nature that produces both specialized nematode-trapping structures and blastospores. Furthermore, *A. blastospora* and *P. dimorphus* share strong similarities with regard to the morphological characteristics of conidia, conidiophores, and the manner of catching nematodes with adhesive materials (Figure 3 and Figure 4). Accordingly, we infer that *A. blastospora* is closely related to *P. dimorphus* and maintained traits of Mesozoic carnivorous fungi, and *P. dimorphus* may be an ancient ancestor of modern Orbiliomycetes carnivorous fungi.

Similar to other fungi, convergent evolution has also been observed in carnivorous fungi; for example, some species of *Dactylellina* (Orbiliaceae, Orbiliomycetes, Ascomycota) and some species of *Nematoctonus* (Agaricomycetes, Pleurotaceae, Basidiomycota) trap nematodes with stalked adhesive knobs [55]. Therefore, we also cannot rule out the possibility of convergent evolution between *A. blastospora* and *P. dimorphus*, which resulted in the sharing of similar characteristics, while having a distant genetic relationship. However, given the scarcity of carnivorous fungi in the fungal kingdom [9,12] and the high similarity of several different structures (conidia, conidiophores, and trapping structures) between *A. blastospora* and *P. dimorphus* (Figure 3 and Figure 4) [23,24], we speculate that it is less likely that these two species would have evolved such similar traits with only a distant genetic relationship.

### 4.3. The Possible Ancestral Position of Palaeoanellus Dimorphus

Among Orbiliomycetes carnivorous fungi, all species are divided into two main groups according to their mechanisms of trapping nematodes. One is the genus *Drechslerella*, which first diverged from other carnivorous species and produces constricting rings to capture nematodes with the mechanical force generated by the expansion of the cells that make up the rings. Another contains all species in the genera *Arthrobotrys* and *Dactylellina*, which catch nematodes with adhesive traps (Figure 1) [11,12,13]. *P. dimorphus* produced unicellular hyphal rings, which possibly produced a sticky secretion used to capture nematodes [23,24]. This structure is similar to those species in *Arthrobotrys* and *Dactylellina* in the manner of trapping nematodes (capture of nematodes performed mainly with adhesive material), but it is quite different from the *Drechslerella* species, which capture nematodes by mechanical force. Therefore, we speculate that *P. dimorphus* is more related to *Arthrobotrys* and *Dactylellina*, and that *P. dimorphus* may be the common ancestor of *Arthrobotrys*, *Dactylellina*, or *Arthrobotrys* and *Dactylellina*.

Generally accepted, modern Orbiliomycetes carnivorous fungi originated from saprophytic fungi without a trapping structure [9,10,11,14,15,16,17]. Their evolution from possessing no trapping structure to complex trapping structures, such as modern adhesive trapping structures, was undeniably a course of gradual complexity. The structural complexity of unicellular adhesive hyphal rings produced by *P. dimorphus* is lower than that of most modern adhesive trapping structures. Therefore, unicellular adhesive hyphal rings may be considered an intermediate stage in the evolution of structural complexity and the common ancestor of all adhesive trapping structures (*Arthrobotrys* and *Dactylellina*). However, based on phylogenetic analysis of multiple genes and molecular clock theory, Yang et al. [9] inferred that the adhesive trapping structures of modern Orbiliomycetes carnivorous fungi originated about 246 million years ago and further evolved around 198–208 million years ago. In contrast, *P. dimorphus* was found in the amber from 100 million years ago [23,24]. Therefore, it can be inferred that the unicellular adhesive hyphal rings produced by *P. dimorphus* are probably not the ancestor of all the modern adhesive-trapping structures (*Arthrobotrys* and *Dactylellina*).

**Figure 6:**
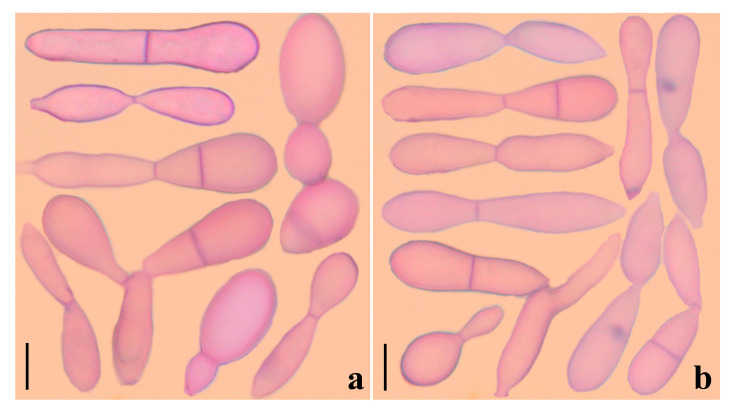
Blastospores of some *Arthrobotrys* species. (**a**) The blastospores produced by *A. oligospora*; (**b**) The blastospores produced by *A. conoides*. Scale bars = 10 μm.

Phylogenetically, *A. blastospora* forms a sister lineage to *A. oligospora* and *A. superba* (Figure 1). Combined with the morphological similarities between *A. blastospora* and *P. dimorphus*, we can infer that *P. dimorphus* is closely related to the *Arthrobotrys* species. Concerning morphology, the following aspects can also support the close relationship between *P. dimorphus* and *Arthrobotrys*: (1) *P. dimorphus* produced unicellular adhesive hyphal rings to capture nematodes [23,24]. This structure is morphologically similar to the single-ring stage of adhesive networks produced by *Arthrobotry* species (Figure 4) [31]. (2) The formation of the unicellular adhesive hyphal rings produced by *P. dimorphus* initiated with a branch which was first generated on the vegetative mycelia, then the branch was curved and fused with the mycelia to form a ring [23,24]. This process is highly similar to the formation process of adhesive networks produced by *Arthrobotrys* species [31] (Figure 4). (3) The blastospores produced by *A. blastospora* are easily separated from each other to form non-septate and 1-septate elliptic conidia. The blastospores produced by *P. dimorphus* also had this characteristic. The non-septate or 1-septate conidia formed by the separation of blastospores are morphologically similar to those of many species in *Arthrobotrys* (Figure 5) [12,31]. (4) Among *Arthrobotrys* species, except *A. blastospora*, a few conidia of *A. oligospora* and *A. conoides* also have a similar morphology to blastospores (Figure 6), which suggests that the formation of blastospores may be an ancestral characteristic of *Arthrobotrys*, or an inherent feature of other *Arthrobotrys* species, but it is rarely developed or not developed in culture and thus, it has been overlooked so far. This phenomenon further illustrated the close relationship between *P. dimorphus* and *Arthrobotrys*.

By contrast, *Dactylellina* demonstrates similarities to *P. dimorphus* only in the aspect of trapping structure: a few species in *Dactylellina* produce a single ring covered with adhesive material (non-constricting ring) to capture nematodes, which is formed by producing a branch from the vegetative mycelia, and then the branch is curved and fused to form a ring [31]. This structure is similar to the unicellular adhesive hyphal rings produced by *P. dimorphus* in the morphology and formation process (Figure 4).

In summary, considering that *P. dimorphus* and *Arthrobotrys* share a reproductive structure (conidia) and nutritional structure (trapping structure) morphology, we speculate that *P. dimorphus* is more likely to be the ancestor of *Arthrobotrys.*

### 4.4. The Necessity of Strengthening the Research on Carnivorous Fungi in the Three Parallel Rivers

The two *A. blastospora* strains were isolated from the core area of the three parallel rivers region in China. This region is located in the southwest of the Heng Duan Mountains where mountains alternate with valleys, the terrain is highly diverse, and the region combines tropical, subtropical, temperate, and alpine cold climate types [56,57]. The complex terrain and climate create rich ecosystems and make the region one of the most biodiverse in the world [58,59]. Glaciers did not cover this region during the Quaternary glaciation due to its unique mountain and deep valley landform, particularly its geographical position, formation and evolutionary process. Therefore, this region is a significant refuge for many ancient species, and it is the center of species distribution and the differentiation of many biological groups [60,61,62,63,64]. According to statistics, 34 species of Chinese national protected plants, 600 species of endemic plants, and 20 species of relict plants are distributed in this region [65,66]. This situation renders it possible to find the relict species of carnivorous fungi in this region and suggests that there may be precious living fossils of other groups living in this region. In addition, *P. dimorphus* was found in the amber from Southwestern France [23,24] and *A. blastospora* was isolated from Southwestern China, more than 11,000 km from each other. This indicates that Palaeoanellus-type fungi were widely distributed and numerous in the past, giving rise to the extant genera of Oribiliomycetes.

## Figures and Tables

**Table 1:** The GenBank accession numbers of the isolates included in this study. The ex-type strains are in bold. The newly generated sequences are indicated in blue.

Taxon	Strain Number	GenBank Accession Number			Reference
		ITS	TEF	RPB2	
***Arthrobotrys amerospora***	**CBS 268.83**	**NR_159625**	**—**	**—**	[36]
*Arthrobotrys anomala*	YNWS02-5-1	AY773451	AY773393	AY773422	[13]
*Arthrobotrys arthrobotryoides*	CBS 119.54	MH857262	—	—	[36]
*Arthrobotrys arthrobotryoides*	AOAC	MF926580	—	—	Unpublished
***Arthrobotrys blastospora***	**CGMCC 3.20940**	**OQ332405**	**OQ341651**	**OQ341649**	**This study**
***Arthrobotrys blastospora***	**ZA173**	**OM956088**	**OQ341650**	**OQ341648**	**This study**
***Arthrobotrys botryospora***	**CBS 321.83**	**NR_159626**	**—**	**—**	[36]
*Arthrobotrys cladodes*	1.03514	MH179793	MH179616	MH179893	Unpublished
***Arthrobotrys clavispora***	**CBS 545.63**	**MH858353**	**—**	**—**	[36]
*Arthrobotrys conoides*	670	AY773455	AY773397	AY773426	[13]
*Arthrobotrys cookedickinson*	YMF1.00024	MF948393	MF948550	MF948474	[12]
*Arthrobotrys cystosporia*	CBS 439.54	MH857384	—	—	[36]
*Arthrobotrys dendroides*	YMF1.00010	MF948388	MF948545	MF948469	[12]
*Arthrobotrys dianchiensis*	1.00571	MH179720	—	MH179826	[37]
*Arthrobotrys elegans*	1.00027	MH179688	—	MH179797	Unpublished
***Arthrobotrys eryuanensis***	**CGMCC** **3.19715**	**MT612105**	**OM850307**	**OM850301**	[32]
*Arthrobotrys eudermata*	SDT24	AY773465	AY773407	AY773436	[13]
*Arthrobotrys flagrans*	1.01471	MH179741	MH179583	MH179845	Unpublished
*Arthrobotrys gampsospora*	CBS 127.83	U51960	—	—	[15]
*Arthrobotrys globospora*	1.00537	MH179706	MH179562	MH179814	Unpublished
*Arthrobotrys guizhouensis*	YMF1.00014	MF948390	MF948547	MF948471	[12]
*Arthrobotrys indica*	YMF1.01845	KT932086	—	—	[38]
*Arthrobotrys iridis*	521	AY773452	AY773394	AY773423	[13]
*Arthrobotrys janus*	85-1	AY773459	AY773401	AY773430	[13]
*Arthrobotrys javanica*	105	EU977514	—	—	Unpublished
***Arthrobotrys jindingensis***	**CGMCC 3.20985**	**OP236810**	**OP272511**	**OP272515**	[39]
***Arthrobotrys jinpingensis***	**CGMCC 3.20896**	**OM855569**	**OM850311**	**OM850305**	[32]
*Arthrobotrys koreensis*	C45	JF304780	—	—	[40]
***Arthrobotrys lanpingensis***	**CGMCC** **3.20998**	**OM855566**	**OM850308**	**OM850302**	[32]
*Arthrobotrys latispora*	H.B. 8952	MK493125	—	—	Unpublished
*Arthrobotrys longiphora*	1.00538	MH179707	—	MH179815	Unpublished
***Arthrobotrys luquanensis***	**CGMCC** **3.20894**	**OM855567**	**OM850309**	**OM850303**	[32]
*Arthrobotrys mangrovispora*	MGDW17	EU573354	—	—	[41]
*Arthrobotrys megalospora*	TWF800	MN013995	—	—	Unpublished
*Arthrobotrys microscaphoides*	YMF1.00028	MF948395	MF948552	MF948476	[12]
*Arthrobotrys multiformis*	CBS 773.84	MH861834	—	—	[36]
*Arthrobotrys musiformis*	SQ77-1	AY773469	AY773411	AY773440	[13]
*Arthrobotrys musiformis*	1.03481	MH179783	MH179607	MH179883	Unpublished
***Arthrobotrys nonseptata***	**YMF1.01852**	**FJ185261**	**—**	**—**	[42]
***Arthrobotrys obovata***	**YMF1.00011**	**MF948389**	**MF948546**	**MF948470**	[12]
*Arthrobotrys oligospora*	920	AY773462	AY773404	AY773433	[13]
*Arthrobotrys paucispora*	ATCC 96704	EF445991	—	—	[13]
*Arthrobotrys polycephala*	1.01888	MH179760	MH179592	MH179862	Unpublished
*Arthrobotrys pseudoclavata*	1130	AY773446	AY773388	AY773417	[13]
*Arthrobotrys psychrophila*	1.01412	MH179727	MH179578	MH179832	Unpublished
*Arthrobotrys pyriformis*	YNWS02-3-1	AY773450	AY773392	AY773421	[13]
***Arthrobotrys reticulata***	**CBS 201.50**	**MH856589.1**	**—**	**—**	[36]
*Arthrobotrys robusta*	nefuA4	MZ326655	—	—	Unpublished
*Arthrobotrys salina*	SF 0459	KP036623	—	—	Unpublished
*Arthrobotrys scaphoides*	1.01442	MH179732	MH179580	MH179836	Unpublished
*Arthrobotrys shizishanna*	YMF1.00022	MF948392	MF948549	MF948473	[12]
***Arthrobotrys shuifuensis***	**CGMCC** **3.19716**	**MT612334**	**OM850306**	**OM850300**	[32]
*Arthrobotrys sinensis*	105-1	AY773445	AY773387	AY773416	[13]
*Arthrobotrys sphaeroides*	1.01410	MH179726	MH179577	MH179831	Unpublished
*Arthrobotrys superba*	127	EU977558	—	—	Unpublished
*Arthrobotrys thaumasia*	917	AY773461	AY773403	AY773432	[13]
***Arthrobotrys tongdianensis***	**CGMCC 3.20942**	**OP236809**	**OP272509**	**OP272513**	[39]
*Arthrobotrys vermicola*	629	AY773454	AY773396	AY773425	[13]
***Arthrobotrys xiangyunensis***	**YXY10-1**	**MK537299**	**—**	**—**	[43]
***Arthrobotrys yunnanensis***	**AFTOL-ID 906**	**DQ491512**	**—**	**—**	[44]
***Arthrobotrys zhaoyangensis***	**CGMCC** **3.20944**	**OM855568**	**OM850310**	**OM850304**	[32]
***Dactylellina appendiculata***	**CBS 206.64**	**AF106531**	**DQ358227**	**DQ358229**	[36]
*Datylellina* *cionopogum*	SQ27-3	AY773467	AY773409	AY773438	[13]
***Dactylellina copepodii***	**CBS 487.90**	**U51964**	**DQ999835**	**DQ999816**	[13]
*Datylellina* *gephyropagum*	CBS178.37	U51974	DQ999847	DQ999802	[13]
*Datylellina haptotylum*	SQ95-2	AY773470	AY773412	AY773441	[13]
*Datylellina haptotylum*	XJ03-96-1	DQ999827	DQ999849	DQ999804	[13]
*Dactylellina leptosporum*	SHY6-1	AY773466	AY773408	AY773437	[13]
***Dactylellina mammillata***	**CBS229.54**	**AY902794**	**DQ999843**	**DQ999817**	[13]
***Dactylellina yushanensis***	**CGMCC** **3.19713**	**MK372061**	**MN915113**	**MN915112**	[45]
*Drechslerella brochopaga*	701	AY773456	AY773398	AY773427	[13]
*Drechslerella dactyloides*	expo-5	AY773463	AY773405	AY773434	[13]
*Drechslerella effusa*	YMF1.00583	MF948405	MF948557	MF948484	Unpublished
*Drechslerella heterospora*	YMF1.00550	MF948400	MF948554	MF948480	Unpublished
*Drechslerella stenobrocha*	YNWS02-9-1	AY773460	AY773402	AY773431	[13]
***Orbilia jesu-laurae***	**LQ59a**	**MN816816**	**—**	**—**	[46]
*Vermispora fusarina*	YXJ02-13-5	AY773447	AY773389	AY773418	[13]

## Data Availability

The data that support the finding of this study are contained within the article.
